# Discovery of a Novel Lineage *Burkholderia cepacia* ST 1870 Endophytically Isolated from Medicinal *Polygala paniculata* Which Shows Potent *In Vitro* Antileishmanial and Antimicrobial Effects

**DOI:** 10.1155/2021/6618559

**Published:** 2021-02-17

**Authors:** Felipe de Paula Nogueira Cruz, Ailton Ferreira de Paula, Camila Tita Nogueira, Paulo Henrique Marques de Andrade, Leonardo Maurici Borges, Paulo Teixeira Lacava, Ilana L B C Camargo, Fernanda de Freitas Aníbal, Cristina Paiva de Sousa

**Affiliations:** ^1^Laboratory of Microbiology and Biomolecules–LaMiB, Department of Morphology and Pathology, Federal University of São Carlos, São Carlos, Brazil; ^2^Laboratory of Inflammation and Infectious Diseases–LIDI, Federal University of São Carlos, São Carlos, Brazil; ^3^Biotechnology Graduate Program, Federal University of São Carlos, São Carlos, Brazil; ^4^Laboratory of Taxonomy and Plant Evolution–TaxEP, Department of Botany, Federal University of São Carlos, São Carlos, Brazil; ^5^Laboratory of Molecular Epidemiology and Microbiology, Physics Institute of Sao Carlos, University of Sao Paulo, São Carlos, Brazil

## Abstract

In this study, we report the isolation and identification of an endophytic strain of *Burkholderia cepacia* (COPS strain) associated with *Polygala paniculata* roots. *Polygala* plants are rich sources of promising microbiomes, of which the literature reports several pharmacological effects, such as trypanocidal, antinociceptive, anesthetic, anxiolytics, and anticonvulsant activities. *B. cepacia* COPS belongs to a new sequence type (ST 1870) and harbors a genome estimated in 8.3 Mbp which exhibits the aminoglycosides and beta-lactams resistance genes *aph*(3′)-IIa and *bla*_TEM-116_, respectively. Analysis performed using MLST, average nucleotide identity, and digital DNA-DNA hybridization support its species-level identification and reveals its novel housekeeping genes alleles *gyr*B, *lep*A, and *pha*C. The root endophyte *B. cepacia* COPS drew our attention from a group of 14 bacterial isolates during the primary screening for being potentially active against *Staphylococcus aureus* ATCC 29213, *Enterococcus faecalis* ATCC 29212, *Micrococcus luteus* ATCC 9341, *Escherichia coli* ATCC 25922, and *Candida albicans* ATCC 10231 and exhibited the broad-spectrum activity against phytopathogenic fungi. In addition, COPS strain showed production of protease, lipase, and esterase in solid media, and its natural product extract showed potent inhibition against fungal plant pathogens, such as *Moniliophthora perniciosa*, whose antagonism index (89.32%) exceeded the positive control (74.17%), whereas *Sclerotinia sclerotiorum* and *Ceratocystis paradoxa* showed high percentages of inhibition (85.53% and 82.69%, respectively). COPS crude extract also significantly inhibited *S. epidermidis* ATCC 35984, *E. faecium* ATCC 700221 (MIC values of 32 *μ*g/mL for both), *E. faecalis* ATCC 29212 (64 *μ*g/mL), and *S. aureus* ATCC 25923 (128 *μ*g/mL). We observed moderate antagonistic activity against *A. baumannii* ATCC 19606 and *E. coli* ATCC 25922 (both at 512 *μ*g/mL), as well as potent cytotoxic effects on *Leishmania infantum* and *Leishmania major* promastigote forms with 78.25% and 57.30% inhibition. In conclusion, this study presents for the first time the isolation of an endophytic *B. cepacia* strain associated with *P. paniculata* and enough evidence that these plants may be considered a rich source of microbes for the fight against neglected diseases.

## 1. Introduction

Plant tissues represent a significant source of natural substances for pharmaceutical and biotechnological interest. Drug discovery has been based on medicinal plants for centuries [1–3]. However, endophytes are capable to biosynthesize a plethora of natural products and compounds which are originally believed to be produced only by their host plants [4, 5], and therefore, they are considered alternative suppliers of characteristic phytochemical compounds and represent a vast unexplored reservoir of unique chemical structures [6]. These plant-symbiont microorganisms that live in intimate interaction establish a mutualistic interaction with the host plant by exchanging nutrients and protection; they produce antibiotics and other substances that can protect the plant against stress conditions such as attack by herbivores, pests, and plant pathogens without causing apparent disease symptoms [7–9].

*Polygala paniculata* (commonly known as “mimosa,” “barba-de-bode,” “barba-de-São-João,” and “vassourinha branca”) is a medicinal plant that frequently grows on the Brazilian coast and is used in traditional medicine due to their analgesic properties and treatment of inflammatory diseases such as asthma, bronchitis, arthritis, and disorders of the kidney [10–12]. However, plants within this genus are well-known producers of a variety of phytochemical compounds [13], such as methyl salicylate, alkaloids [14], xanthones [15, 16], saponins [17, 18], coumarins [11, 19], and styrylpyrones [11]. Natural products extracted from *Polygala* species are widely studied [13], and numerous reports describe pharmacological effects for their crude extracts such as anti-inflammatory [20, 21], anxiolytic [22], antidepressant [11], trypanocidal [23], antinociceptive [24], neuroprotective [25, 26], antiatherosclerosis [27], antitumor [28, 29], and antifungal [30]. However, the potential of endophytes and rhizospheric-associated microorganisms within these plant genuses remains unknown. Consequently, the exploitation of medicinal plants' microbiome, which produces bioactive metabolites, is fundamental [31, 32].

The introduction of antibiotics enabled the development of therapies for previously incurable diseases. However, resistance to this class of medicines happens faster than the human capability of discovering new compounds and introducing them into clinical practice. Moreover, synthetic approaches to antibiotic production have not been effective enough to completely replace this platform [31, 33, 34].

Likewise, phytopathogenic fungi represent a severe threat to several crops, thus affecting production and quality. Modern agriculture is entirely dependent on agrochemicals; although they can improve crop yield, quality, and shelf-life, they negatively affect the environment and human health. In this regard, issues related to sustainability and practices in defense of the environment have drawn considerable attention [35–37].

Approximately, 13 million people suffer from parasitic diseases caused by *Leishmania* protozoa infection. Parasite resistance and host toxicity of currently available drugs are a reality and a concern mainly in subtropical countries [38, 39]. On the other hand, microbial resistance to antibiotics has been rapidly spread, is responsible for 33,000 deaths in Europe, and became a concern to public health [40].

We aimed to explore the endophytes of *Polygala paniculata* and isolate antibiotic- and biotechnology-related enzymes-producing microorganisms. Herein, we present the *Burkholderia cepacia* COPS, a sequence-type (ST) 1870 strain isolated from *P. paniculata* roots collected in the Brazilian Atlantic Forest. In addition to its draft-genome, we presented the COPS enzymes and antimicrobial activities.

*Burkholderia* spp. consist of emerging sources of a plethora and diverse natural products potentially relevant for therapeutic/medicine, biotechnological, and agriculture applications [41]. This genus is a versatile producer of antimicrobial compounds and enzymes and exhibits plant growth-promoting properties. We can find such Gram-negative bacteria in several habitats, ranging from humans (as pathogens) to plants (as endophytes) [42–48].

Although widely used for bacterial systematics, the taxonomic identification by 16S rRNA coding gene among *Burkholderia cepacia* complex (Bcc) is limited and difficult [49–53]. According to Bach et al. [52], the identities of 16S rRNA and *recA* genes within Bcc can reach 100% and 95%, respectively. Several studies report the use of housekeeping genes established in multilocus sequence typing (MLST) to differentiate the Bcc species [54–61]. The multilocus sequence analysis (MLSA) of the housekeeping genes *atp*D, *glt*B, *gyr*B, *rec*A, *lep*A, *pha*C, and *trp*B enable an accurate investigation of evolutionary characteristics and high resolution at the species level [56, 57, 61].

## 2. Materials and Methods

### 2.1. Biological Material

*Polygala paniculata* plants samples and their rhizospheric soil were collected in Peruíbe, south coastal municipality of São Paulo State, Brazil (−24° 19 ′12 ″, 46° 59′ 54″) (SisGen, registration number: AF1A75A), and vouchers (F.P.N. Cruz 3) were deposited at the Federal University of Sao Carlos (SPSC) and Botanical Garden of Rio de Janeiro (RB) herbaria and registered to the Brazilian Genetic Resources Managing System (SisGen; registration AF1A75A). Plant's identity was confirmed as *Polygala paniculata* Linnaeus [62], in accordance with Marques and Gomes [63]. The bacterial community was isolated after superficial disinfection of the plant by serial washing in 70% ethanol for 2 min, NaClO for 3 min, 70% ethanol for 1 min, and double rinse with distilled H_2_O. The plant structures were grounded and incubated in phosphate-buffered solution (PBS) (NaCl, 8.0 g/L; KCl, 0.2 g/L; Na_2_HPO_4_, 1.44 g/L; KH_2_PO_4_, 0.24 g/L; pH, 7.4) at 28°C/200 rpm for 2 hours. Subsequently, 100 *μ*L of 1 : 10 serial dilutions were inoculated on tryptic soy agar (TSA) supplemented with benomyl (50 *μ*g/mL) and incubated at 28°C until growth [64, 65]. The isolation of the rhizospheric community was based on Andreote et al. [66] with slight modifications. Ten grams of rhizospheric soil were placed in Erlenmeyer flasks containing 90 mL of sterile PBS and incubated under the same conditions already described. Finally, 100 *μ*L of decimal dilutions was inoculated in TSA and cultured at 28°C until bacterial growth.

### 2.2. Screening of Antimicrobial and Enzymatic Activities

Fourteen bacterial isolates were randomly selected (four from rhizosphere and ten endophytes) and qualitatively tested by the overlay test [44, 67, 68] for the antimicrobial activity screening. A total of 100 *μ*L of precultured isolates in International *Streptomyces* Project medium 2 (ISP2) (malt extract: 10 g/L; yeast extract: 4 g/L; and glucose: 4 g/L) [69] was adjusted to OD_600_ between 0.3 and 0.6 and inoculated in the center of the Petri dishes containing ISP2 agar and incubated at 28°C for 72 hours. Then, the isolates were exposed to chloroform for inactivation, followed by a 30-minute evaporation step. Subsequently, semisolid brain-heart infusion agar (BHI), previously inoculated with test microorganisms (*Staphylococcus aureus* ATCC 29213*, Escherichia coli* ATCC 11775, and *Candida albicans* ATCC 10231), was poured onto the inactivated isolate.

The antagonism test for phytopathogenic fungi was performed as described by Quiroga et al. [70]. The isolated strains were streaked in potato dextrose agar (PDA) at the edges of the plates and incubated at 28°C until their complete growth. Next, a 0.6 cm plug of the phytopathogenic fungus mycelium was placed at the top of the plate containing the grown isolate. The plates were incubated again at 28°C. As a negative control, each phytopathogen was cultured in PDA for indicating the time of the inhibition evaluation [71]. All tests were performed in triplicates. All pathogenic strains used in this study are listed in Table 1.

The assessment of enzymatic potential consisted in a preculture of *Polygala paniculata*-derived bacteria in 3 mL of tryptic soy broth (TSB–KASVI) and incubated for 48–72 hours at 28°C. Then, 2 *μ*L of the culture was transferred to a M9 enzymatical solid medium (200 mL/L of stock solution (64 g/L Na_2_HPO_4_.7H_2_O; 15 g/L KH_2_PO_4_; 2.5 g/L NaCl; 5 g/L NH_4_Cl)); 2 mL/L 1 M MgSO_4_; 10 g/L; 0.1 mL/L CaCl_2_ 1 M; 15 g/L agar, pH 7.2, with different supplements, depending on the activity to be studied: (1) 0.5% yeast extract and 1% soluble starch for amylase activity; (2) 0.5% yeast extract and 1% carboxymethyl cellulose for cellulase activity; (3) 0.5% yeast extract and 1% pectin, pH 8.0 for pectin-pectate lyase; and (4) 0.5% yeast extract and 1% pectin, pH 5.0 for pectin-polygalacturonase. The lipase/esterase media consisted of peptone, 10 g/L; NaCl, 5 g/L; CaCl_2_.H_2_O, 0.1 g/L; agar, 15 g/L; pH 7.4, supplemented with 1% (v/v) of Tween 20 and Tween 80 for lipolytic and esterastic activities, respectively. The following components were used for protease medium: 5 g/L of tryptone; 2.5 g/L of yeast extract; 1.0 g/L of glucose; 2.5 g/L of NaCl; 15 g/L of agar; and the pH adjusted to 7.0. All components were sterilized at 121°C for 15 minutes, and 100 mL skimmed milk was added for completing one liter.

The experiment was performed in triplicate, and the isolates were incubated for 48 h at 28°C. Congo red dye was used as a revealer (15 minutes) followed by a washing step with 5 M NaCl for the cellulase activity visualization. Iodine tincture was used for amylase and pectinases tests. The enzymatic production of protease, lipase, and esterase activities was visualized as a bright halo around the colonies [72].

### 2.3. Natural Products Extraction

The isolate GLB 2 was selected for the next experiments due to its broad-spectrum, high bioactivity rates in antimicrobial screening, and capacity to biosynthesize multiple enzymes. The natural products extract (NPE) of the isolate GLB 2 was obtained through the inoculation of 10 *μ*L of a preculture in round-bottom tubes (12 mL capacity) containing 3 mL of ISP2 and incubated at 220 rpm/28°C for three days. Subsequently, the culture was inoculated in 100 mL of ISP2 in a 250 mL flask and maintained under the same conditions for seven days. The culture was then centrifuged at 4,500 rpm for 10 min and extracted by solid-phase using polypropylene mesh packages containing 1.5 g of Amberlite® XAD16 resin (Sigma-Aldrich), which were added to the fermented broths and overnight incubated on a rotary shaker under the same conditions described. The resin bags were then removed and packed in glass tubes containing 20 mL of MeOH : EtOAc (1 : 1). The extracts were dried and concentrated by Vacufuge plus (Eppendorf), resuspended at a 50 mg/mL concentration in 100% dimethyl sulfoxide (DMSO) and maintained at −80°C [73].

### 2.4. Antagonism Index in Phytopathogens

**Figure 1:**
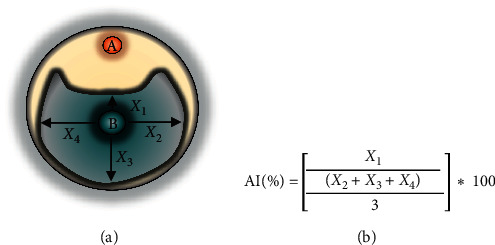
Schematic representation of the quantitative assay for the AI determination: (a) paper disk containing NPE of *Burkholderia cepacia* COPS; (b) phytopathogenic fungus mycelium. The inhibition index was calculated by the formula using the means of the mycelial growth measurements in centimeters.

The isolate GLB 2 NPE antagonism index (AI) was determined by the inoculation of 500 *μ*g of its NPE in a sterile disk placed on top of the plate. A plug of phytopathogenic fungus was inoculated in the center. The assay positive control consisted of culturing each phytopathogen, as described above, in the presence of 500 *μ*g of benomyl, whereas for the negative control, each phytopathogen was cultured to indicate the expected fungi growth. All measurements were performed in triplicate (Figure 1).

### 2.5. Minimum Inhibitory Concentration

The minimum inhibitory concentration (MIC) of the NPE was performed in triplicate, according to recommendations of the Clinical and Laboratory Standards Institute (CLSI) [74] against pathogenic bacteria *Staphylococcus aureus* ATCC 25923, *Staphylococcus epidermidis* ATCC 35984, *Enterococcus faecalis* ATCC 29212, *Enterococcus faecium* ATCC 700221, *Acinetobacter baumannii* ATCC 19606, *Escherichia coli* ATCC 25922, *Klebsiella pneumoniae* ATCC 700603, and *Pseudomonas aeruginosa* ATCC 27853. An overnight culture of each pathogen was diluted until reaching a 5 × 10^5^ CFU.mL^−1^ final concentration in Müeller–Hinton cation adjusted (MHCA) broth with several concentrations of COPS NPE (from 100 to 1.5 *μ*g/mL at 1% DMSO) and incubated for 24 h, at 37°C. As a positive control, ciprofloxacin was used at the same concentration gradient, and bacterial suspensions in MHCA broth and 1% DMSO were used as negative controls. The bioactivity was analyzed by measuring each well's optical density 24 hours after administration of NPE in a microtiter plate reader.

### 2.6. In Vitro Activity Assay against *Leishmania* spp

Cultures of promastigote forms of *Leishmania infantum* strain MHOM/BR/1972/LD and *L. major*, maintained at −80° C in a freezing solution (DMSO/fetal bovine serum—1 : 10), were thawed and transferred to 9 mL of Schneider's medium (Sigma-Aldrich, USA) supplemented with 10% inactivated fetal bovine serum (Vitrocell Embriolife, BRA), 10% human urine from male volunteers aged between 25 and 35 years, and 1% of penicillin and streptomycin. The cultures were then centrifuged for 5 min at 5000 rpm, and the pellet was resuspended in 1 ml of the same medium, which was transferred to a 50 mL capacity cell culture bottle containing 9 mL of fresh medium and incubated at 26°C in 5% CO_2_.

The toxicity assay consisted of the inoculation of promastigotes in the stationary phase (10^7^ cells/mL) in 96 well plates containing different concentrations of NPE (200, 100, 50, 25, 10, and 1 *μ*g/mL). They were tested in biological triplicates and experimental duplicates. The OD_550_ was measured by a spectrophotometer (Thermo Scientific Multiskan GO spectrophotometer) 24 hours after the administration of NPE. Amphotericin B (Sigma-Aldrich, USA) at 100 *μ*M was used as a positive control. The cell viability percentage was calculated from the absorbance of the negative control, which represents 100% of cell viability (% of living cells = test OD_550_ × 100/negative control OD_550_), and IC_50_ was measured by nonlinear regressions of the values found for each concentration in, at least, three independent experiments.

### 2.7. Statistical Analysis

The results were analyzed by GraphPad Prism 8.0.1 software (San Diego, California, USA), and the Shapiro–Wilk test was applied to all data obtained. Subsequently, one-way ANOVA (one-way analysis of variance), followed by Dunnett's multiple comparisons test were applied using a statistical significance at *p* < 0.05 (95%).

The statistical significance in *Leishmania* assays was calculated by Tukey's multiple comparison test after one-way ANOVA analysis.

### 2.8. Genomic DNA Isolation

Isolate GLB 2 was cultivated for three days/28°C in 20 mL of ISP2 broth (malt extract, 10 g/L; glucose, 4 g/L; yeast extract, 4 g/L; pH 7,3), and cells were harvested by centrifugation at 8000 rpm for 10 minutes. Genomic DNA was then extracted using DNeasy Kit (Qiagen) with a lysis process consisting of four cycles of incubation of cell pellets at 65°C for 15 minutes in 180 *μ*L of ATL buffer followed by freezing at −80°C for 15 minutes. DNA was eluted in 100 *μ*L of H_2_O, and its quality was analyzed on 0.7% agarose gel stained with hydra green and quantified using a NanoDrop.

### 2.9. Genome Sequencing, Assembly, and Functional Annotations

Pair-ended sequences were obtained by Illumina MiSeq (Illumina, San Diego, USA) platform using a 2x250 bp library prepared using Nextera XT DNA kit with v3 600 cycles. Illumina paired end reads were first preprocessed for quality analysis using FastQC—Unipro UGENE v. 34 [75] and trimmed using Trimmomatic—Galaxy v. 0.38.0 [76] for removing quality bases lower than 20 and adapters. *Burkholderia* sp. genome was assembled using SPAdes Genome Assembler—Galaxy v. 3.12.0 [77] applying kmers 21, 33, and 55. Quality assessment of assemblies was evaluated using the software QUAST Genome assembly Quality—Galaxy v. 5.0.2 [78] followed by annotation using Prokka—Prokaryotic genome annotation v. 1.14.1—Galaxy v. 1.14.5 [79] and Rast v. 2.0 [80] (https://rast.nmpdr.org/rast.cgi). Finally, GLB 2 genomic contigs were mapped against the reference genome of *B. cepacia* ATCC 25416 using CONTIGuator v. 2.7.4 [81].

### 2.10. Phylogenetic and Multilocus Sequence Analysis

Isolate GLB 2 16S ribosomal RNA gene was identified by RNAmmer 1.2 server (http://www.cbs.dtu.dk/services/) [82] and identified based on 16s rRNA BLAST search.

The complete 16S rRNA and housekeeping gene sequences *atp*D (ATP synthase *β* chain—1,395 bp), *glt*B (glutamate synthase large subunit—4,704 bp), *gyr*B (DNA gyrase B—2,475 bp), *rec*A (recombinase A—1,071 bp), *lep*A (GTP binding protein—1,794 bp), *pha*C (acetoacetyl-CoA reductase—741 bp), and *trp*B (tryptophan synthase subunit B—1,194 bp) from reference Bcc members were retrieved from PubMLST database (http://www.pubMLST.org/bcc/) and aligned with the endophytic *B. cepacia* nucleotide sequences by ClustalW and concatenated using MEGA X software [83].

The phylogenetic trees of multiple alignments of 16S rRNA and concatenated housekeeping gene sequences were generated by MEGA X. The neighbor-joining method [84, 85] using Jukes-Cantor as a substitution model, respectively, as well 1000 bootstrap replications as branch support for the construction of the trees.

### 2.11. Comparative Genomic Analysis

Average nucleotide identity (ANI) [86–88], calculation of tetra nucleotide frequencies, and correlation coefficients [89] values were estimated based on BLAST alignments using JSpeciesWS (http://jspecies.ribohost.com/jspeciesws/) [90, 91], whereas GGDC 2.1 (http://ggdc.dsmz.de/distcalc2.php) using BLAST + alignment and recommended formula (2) were used to calculate the genome-to-genome distance [92, 93].

Additional in silico analyses of GLB 2draft-genome were performed using PathogenFinder 1.1 [94] and ResFinder 4.1 [95, 96] to estimate the number of pathogenicity determinants and antibiotic resistance genes (https://cge.cbs.dtu.dk/services).

## 3. Results

### 3.1. Antimicrobial and Enzymatic Potential Screening

Fourteen bacterial isolates were randomly selected (endophytes and from rhizosphere) and tested by overlay assay against *S. aureus* ATCC 29213, *E. coli* ATCC 11775, and *C. albicans* ATCC 10231 and phytopathogenic fungi as a primary selection on solid media. The results of the antimicrobial and enzymatic screening assay data are summarized in Tables 2 and 3. We observed that protease was the most abundant enzymatic activity detected, followed by pectinase at pH 8.0 (pectate lyase). Based on such results, we selected the isolate GLB2, which showed the antagonism activity against all tested pathogens and presented the enzymatic activity. We coined the isolate *Burkholderia cepacia* COPS strain in this study.

### 3.2. *Burkholderia cepacia* COPS Genome

Root endophyte GLB2 isolate, which revealed a broad-spectrum and potent activity against all pathogens tested, was identified as *Burkholderia cepacia* based on 16s rRNA BLAST (GenBank accession number MN939546) search. The phylogenetic analysis revealed the isolate belongs to the *Burkholderia* sensu stricto group and is closely related to Bcc genomovar.

Genome sequencing of *Burkholderia cepacia* COPS strain generated 1,970,487 reads with an average length of 35–251 bp. The assembled genome estimated in 8.3 Mbp distributed in 80 contigs with an N50 of 275,353 bp. The functional annotation predicted 1885 genes, of which 1,838 are protein-coding genes (CDSs), 21 tRNAs, 26 misc RNAs, and 66.87% of a GC content. The draft-genome sequence was deposited in GenBank under accession number WIXR00000000.

Phylogenetic inferences (neighbor-joining and maximum likelihood) of COPS strain and Bcc reference strains of individual 16S rRNA and housekeeping genes *atp*D and *pha*C showed low-resolution bootstrap values. In addition, COPS strain is clustered separately from *B. cepacia* UCB 717 forming a single branch, whereas *glt*B, *gyr*B, *lep*A, *rec*A, and *trp*B were strongly supported, as well lineages clearly grouped (Supplementary Material 1–8).

**Figure 2:**
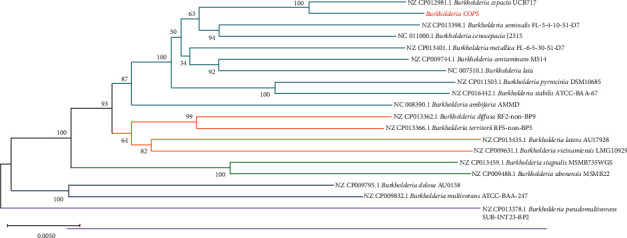
Consensus tree obtained from a neighbor-joining phylogenetic analysis using the Jukes-Cantor method (bootstrap with 1000 replicates) based on the concatenated full length of seven housekeeping and 16S rDNA gene of *B. cepacia* COPS strain compared to reference sequences of Bcc members.

The multilocus sequence typing of *Burkholderia cepacia* COPS using the MLST-2.0 server revealed this strain belongs to the novel ST 1870 (ID 3851), with the following alleles numbers: *atp*D (235), *glt*B (707), *trp*B (21), *rec*A (1), *gyr*B (1205), *lep*A (789), and *pha*C (607). Phylogenies regarding concatenated sequences (14833 bp) of the full length of seven housekeeping genes showed a significant branch support and strong association with *B. cepacia* UCB717 (Figure 2).

Pairwise digital DNA-DNA hybridization (dDDH) of COPS strain compared to 13 sequences of other *Burkholderia cepacia* strains revealed identity levels ranging from 87.90% to 80.70%, whereas ANIm, ANIb, and tetranucleotide frequency signature (TETRA) values of *Burkholderia* sp. LK4, *B. reimsis* BE51, and *B. lata* LK27 were interestingly slightly higher when compared to *B. cepacia* strains.  Table 4 summarizes the genome level comparisons of COPS to other *B. cepacia* strains.

Genome analysis performed using ResFinder 4.1 revealed the presence of ORFs corresponding to aph(3′)-IIa (99.75% identity) and blaTEM-116 (100% identity) genes, which confer resistance to aminoglycosides and beta-lactams, respectively. Nevertheless, *B. cepacia* COPS was estimated in 0.829 as a human pathogen, and its genome matched 53 pathogenic and five nonpathogenic families by PathogenFinder 1.1.

### 3.3. Antagonistic Effects of *Burkholderia cepacia* COPS Crude Extract

**Figure 3:**
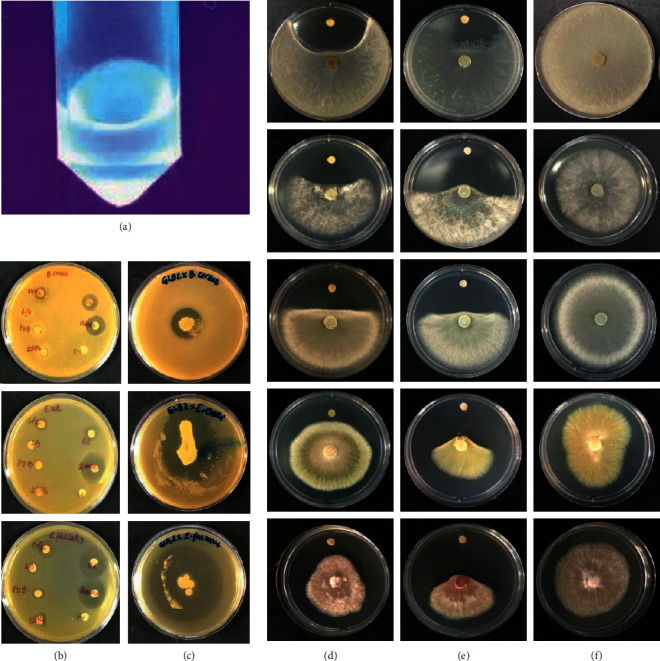
Antimicrobial activity of NPE (a) produced by *B. cepacia* COPS. Columns (b) and (c) correspond to different crude extracts' antibacterial activity compared to overlay assay (top to bottom: *Bacillus cereus*, *Escherichia coli,* and *Enterococcus faecalis*). Lanes (d) COPS NPE, (e) benomyl, as positive control, and (f) negative control represent the antifungal effect. From top to bottom: *Moniliophthora perniciosa, Ceratocystis paradoxa*, *Sclerotinia sclerotiorum*, *Alternaria alternata*, and *Fusarium verticillioides.*

Regarding the bioactivity against bacterial and yeast pathogens in overlay assay, *B*. *cepacia* COPS potently inhibited *S. aureus* ATCC 29213, *E. faecalis* ATCC 29212, *E. coli* ATCC 25922, *M. luteus* ATCC 9341, and *C. albicans* ATCC 10231. However, the NPE obtained from the COPS cultivation in ISP2 revealed inhibition against Gram-positive bacteria as well as *A. baumannii* ATCC 19606 and *E. coli* ATCC 25922 at 512 *μ*g/mL. In addition, the NPE inhibited all phytopathogenic fungi in primary screening by a two-by-two streaking test. The inhibition index calculation was based on the inoculation of 500 *μ*g/disk of *B. cepacia* COPS NPE. The phytopathogenic fungi *M. perniciosa*, *S. sclerotirium,* and *C. paradoxa* showed the highest inhibition percentages (89.32%, 85.53%, and 82.69%, respectively). The antagonism activity of NPE exceeded the positive control (74.17%) of *M. perniciosa*, whereas *R. microsporus* and *F. oxysporum* ATCC 2163 were less inhibited (6.59% and 7.17%, respectively) (Figure 3 and Table 5).

### 3.4. Activity against *Leishmania* spp

We tested the activity of *B. cepacia* COPS NPE *in vitro* against *Leishmania infantum* and *Leishmania major*. A 24-hour treatment with NPE concentrations ranging from 200 to 1 *μ*g/mL potentially inhibited *L. infantum* promastigotes with cell viability ranging from 27% to 78.25%, respectively, with IC_50_ of 86.6 *μ*g/mL. Moreover, the NPE in the highest concentration tested was more effective than amphotericin B at 100 *μ*M (28.6% cell viability).

**Figure 4:**
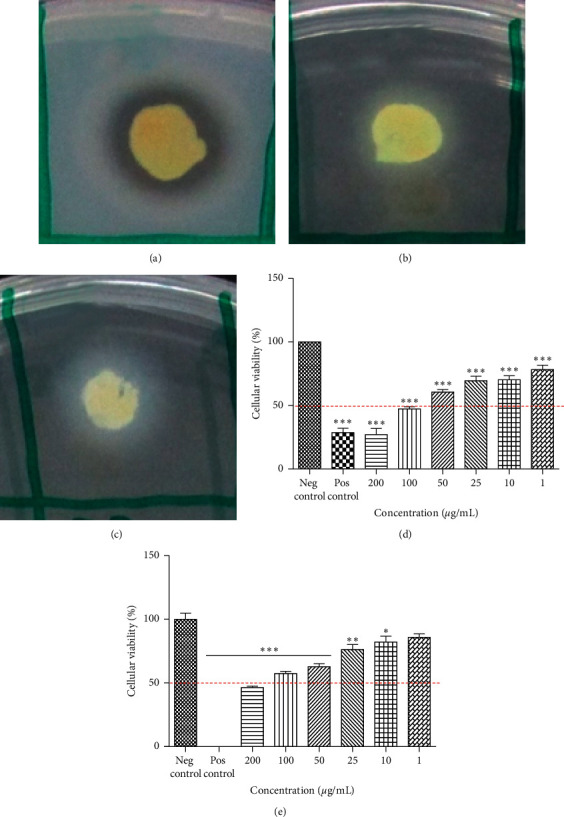
Evaluation of enzymatic production of *Burkholderia cepacia* COPS in solid media: (a) protease; (b) esterase; (c) lipase. Activity of *B. cepacia* COPS NPE at different concentrations against promastigotes of *L. infantum* (d) and *L. major* (e) after 24 hours of exposure. Values represent two independent experiments performed in triplicate. ^*∗∗∗*^Indicates statistical significance compared to the negative control, with values of *p* < 0.001.

On the other hand, the effect of COPS NPE against *Leishmania major* was not so potent compared to *L. infantum*. The results show 57.30% cell viability in a 200 *μ*g/mL concentration and an IC_50_ of 94.55 *μ*g/mL.  Figure 4 summarizes the enzymatic and antileishmanial effects of *B. cepacia* COPS.

## 4. Discussion

*Burkholderia* spp. are nonfermenting bacteria spread worldwide and highly able to adapt to various ecological niches [43, 46, 97–99]. Although Bcc is considered potentially life-threatening as an opportunistic pathogen in hospital environments, especially in patients with cystic fibrosis, *Burkholderia* spp. are versatile for biotechnological applications, and their potential has been exploited for bioremediation, plants growth promotion, biological control, and broad-spectrum agents in several members of this group [41, 46, 47, 100].

*Burkholderia* genus (sensu lato) comprises more than 100 species that possess the ability to adapt to various ecological niches [99, 101]. Members of this complex group exhibit genomes ranging from 7 to 9 Mbp, resulting in several taxonomic rearrangements [46, 99], rapid mutation, and adaptation [102]. Bcc species exhibit high genetic similarity and is phenotypically indistinguishable [102–104], leading to often misidentifications interfering the effective treatment and epidemiological studies [52, 105].

Our analyses concerning COPS strain for pathogenicity determinants using PathogenFinder 1.1 revealed a prediction of pathogenic potential of 0.829, whereas the reference *B. cepacia* ATCC 25416 was 0.774. Interestingly, Z-scores of TETRA correlation were slightly higher (*Burkholderia* sp. LK4, *B. reimsis* BE51, and *B. lata* LK27) when compared to *B. cepacia* strains.

The analysis of sequence variations of *rec*A and *his*A genes offers further discriminatory support at species-level identification [53, 104, 106]. However, multilocus sequence typing offers more sensitivity to identify species within Bcc [57, 107–109]. Our results showed a significant branch support (100%) and a strong association with *B. cepacia* UCB717 and revealed novel alleles of *gyr*B, *lep*A, and *pha*C, which led us to describe the novel ST 1870. As mentioned, *Burkholderia* spp. may be found in a wide range of niches, and genetic variations might occur in response to niche adaptation [41, 109].

Regarding the individual alignments of *atp*D and *pha*C, our results showed low-resolution bootstrap values, which corroborate inconsistencies found in MLST, ANI, and DNA hybridization of the soil isolate *Burkholderia catarinensis* sp. nov., formerly reported as *B. cepacia* [110]. Analysis by matrix-assisted laser desorption/ionization time-of-flight mass spectrometry (MALDI-TOF MS) enabled the proper identification of novel species of *Burkholderiales*. Interestingly, *B. catarinensis* exhibits physiological characteristics that differ from most other Bcc species [52].

In summary, the present study confirms that COPS strain is closely related to *B. cepacia* based on the whole-genome ANI, individual housekeeping gene sequence analysis, and MLST. Moreover, we present for the first time the identification of a *B. cepacia* symbiotically/endophytically associated with *Polygala paniculata* suggesting that *Polygala* genus plays a role in harboring microorganisms for biotechnological applications.

The detection of *in vitro* production of active compounds may be tricky. Usually, quorum sensing (QS) also controls the production of multiple antimicrobial substances. The cell density can promote a signaling system that transcribes certain genes for their interaction with their hosts and increases resistance to stresses [43, 111]. However, the presence of nutrients in the fermentation medium might affect the biosynthesis of QS signaling molecules, such as N-acyl homoserine lactones. Keum et al. [112] demonstrated that glucose (present in ISP2 at 4 g/L in its composition) increases the biomass, but suppresses the production of pyrrolnitrin, which is effective against fungi, yeasts, and Gram-positive bacteria [113]. Nevertheless, Figure 4 shows that the COPS NPE produced by the cultivation in ISP2 still exhibited potent inhibition against phytopathogenic fungi (lanes D, E, and F). Interestingly, regarding cell proliferation, the cultivation of *B. cepacia* COPS in ISP2 showed higher cell density comparing to other media, such as Luria–Bertani medium [114], PDB [67], and 2S4G [115]. However, in disk diffusion assay, the NPEs obtained by cultivation in different media presented a nonsignificant activity (lane B). In contrast, the overlay assay using ISP2 showed a moderate activity against *B. cereus* and higher inhibition zones against *E. coli* and *E. faecalis* (lane C). Thus, these data corroborate related studies [112, 116, 117] concerning multiple secondary metabolites' production.

In agrobiology, the broad-spectrum activity of microorganisms that inhibit plant pathogens is critically important for biological control because the ability to antagonize phytopathogens can indirectly promote the host plant growth [99, 118]. In this context, Orlandelli et al. [118] evaluated antagonism and competitive interactions of endophytic fungi isolated from *Piper hispidum* against *Alternaria alternata*, *Colletotrichum* sp., *Phyllosticta citricarpa*, and *Moniliophthora perniciosa*. Although fungus *Lasiodiplodia theobromae* showed activity against all tested phytopathogens in a dual culture assay, it exhibited antagonism indexes of 60.09% against *M. perniciosa*, 64.79% in *A. alternata*, and 54.16% in *Colletotrichum* sp. In our study, the NPE of *Burkholderia cepacia* COPS showed better inhibition toward *M. perniciosa*, thus exceeding the positive control (74.17%). On the other hand, the COPS crude extract showed mild effects toward *A. alternata* (22.53%) and *Colletotrichum* sp. (42.27%). The phytopathogenic fungus *Moniliophthora perniciosa*, which causes the witches' broom disease in cacao crops, is responsible for 90% losses in the cacao annual production [119, 120]. Therefore, it is of interest to have a microorganism, such as *B. cepacia* COPS, that could help in the cacao crops infection control.

Interestingly, de Almeida Lopes et al. [121] isolated three endophytic strains of Bcc from soybean plants. The cultivation in nutrient broth produced bioactive lipopeptides, extracted by different methods (methanol, ethyl acetate, and ammonium sulfate precipitation), and qualitatively tested regarding their capacity to inhibit fungal (*S. sclerotiorum*, *P. sojae*, and *R. solani*) and bacterial (*X. axonopodis* pv. *glycines* and *P. savastanoi* pv. *glycinea*) plant pathogens. The inhibition rates against the phytopathogenic fungi exceeded 70%; whereas, in our study, 200 *μ*g of the COPS NPE promoted a potent inhibition of *Sclerotinia sclerotiorum* and showed a moderate activity against *P. sojae*.

Our analysis revealed that COPS NPE can potentially inhibit *C. paradoxa* but exhibits moderate and slight activities against *Colletotrichum* sp. and *Fusarium verticillioides*, respectively. Such phytopathogenic fungi and others are responsible for high losses in the production of several crops worldwide [122, 123]. C*. paradoxa* causes the black rot postharvest disease in pineapple [124] and also infects sugarcane [125, 126], palm trees, cacao plants, and several other crops [127]. *F. verticillioides* is a producer of fumonisin, a carcinogenic mycotoxin [128], and other species have been described as emergent and opportunistic pathogens in humans [129]. Fávaro et al. [125] monitored *E. nigrum* endophytically inoculated in sugarcane plants, and its extract significantly reduced the diameter of *Fusarium verticillioides*, *Colletotrichum falcatum*, *Ceratocystis paradoxa*, and *Xanthomonas albilineans* colonies at concentrations ranging from 0.1 to 2.0 mg/mL.

As discussed above, the COPS strain was capable of producing lipase in solid medium, and its NPE potentially inhibited *L. infantum* and *L. major*. These observations corroborate the work of Alves et al. [130] that investigated the antileishmanial activity of crude extracts of lipase-producing endophytic fungi toward *Leishmania amazonensis*. The antileishmanial activity of lipases of *Vermisporium* sp. (78.88%), *Emericella nidulans* (39.65%), *Dichotomophtora portulacae* (63.17%), and *Dichotomophtora boerhaaviae* (98.13%) was detected at 5 mg/mL in amastigote forms, suggesting an enhancement of antileishmanial activity by lipases due to their thermal stability and resistance to several organic solvents, including alcohols [131]. Therefore, a detailed analysis of compounds produced by *B. cepacia* COPS is fundamental for a complete understanding of its potent antileishmanial effect.

Considering that the leishmaniasis treatment with pentavalent antimonials is known to be ineffective and unsafe and therapies based on pentamidine and amphotericin B are considered toxic and exhibit recurrence rates, it is clear that new treatment alternatives are necessary [132–136]. Although widely described as a producer of antibacterial and antifungal compounds [41, 42, 137–139], we emphasize that the *Burkholderia* species' antileishmanial activity was unknown so far. As we demonstrated in this study, *Burkholderiales* may offer promising candidates to treat neglected diseases of which resistance and toxicity of current treatments represent a global public health concern.

## 5. Conclusions

This research's novelty lies in the isolation of rhizospheric and endophytic bacteria associated with *Polygala paniculata* and isolation and biological activity determination of the root endophyte *Burkholderia cepacia* COPS strain. Our results demonstrated that *Polygala paniculata* is a promising source of microorganisms for the fight against *Leishmania* spp., bacteria of clinical importance, and phytopathogens.

## Figures and Tables

**Table 1:** Pathogenic strains used in this study.

Public health pathogens	Phytopathogens
*Staphylococcus aureus* ATCC 25923	*Sclerotinia sclerotiorum*
*Staphylococcus aureus* ATCC 29213	*Moniliophthora perniciosa*
*Staphylococcus epidermidis* ATCC 35984	*Fusarium solani*
*Enterococcus faecium* ATCC 700221	*Fusarium oxysporum* ATCC 2163
*Enterococcus faecalis* ATCC 29212	*Sphaceloma* sp.
*Micrococcus luteus* ATCC 9341	*Ceratocystis paradoxa*
*Acinetobacter baumannii* ATCC 19606	*Alternaria alternata*
*Escherichia coli* ATCC 25922	*Fusarium proliferatum*
*Klebsiella pneumoniae* ATCC 700603	*Colletotrichum* sp.
*Pseudomonas aeruginosa* ATCC 27853	*Fusarium verticillioides*
*Candida albicans* ATCC 10231	*Fusarium oxysporum—*bean
	*Fusarium oxysporum—*cotton
	*Phytophthora sojae*
	*Rhizopus microsporus*

**Table 2:** Results of the initial screening against pathogens.

	Pv110	Pv168	GLB10″	GLB10′	Roxo20	Pv150	Pv48	Roxo19	Roxo16	Pv55	WFRh72	Pv46	Unk3	GLB2
*Human pathogens*
*Escherichia coli* ATCC 25922	−	−	−	−	−	−	−	−	−	+	−	−	−	+
*Staphylococcus aureus* ATCC 29213	−	−	−	−	+	−	−	+	+	−	−	−	+	+
*Candida albicans* ATCC 10231	−	−	−	−	−	−	−	−	−	−	−	−	−	+

*Phytopathogenic fungi*														
*Moniliophthora perniciosa*	−	−	−	−	−	−	−	−	−	−	−	−	+	+
*Sclerotinia sclerotiorum*	−	−	−	−	−	−	−	−	−	+	−	−	+	+
*Fusarium solani*	−	−	−	−	−	−	−	−	−	+	−	−	+	+
*Fusarium verticillioides*	−	−	−	−	−	−	−	−	−	+	−	−	+	+
*Fusarium proliferatum*	−	−	−	−	−	−	−	−	−	+	−	−	+	+
*Fusarium oxysporum* (bean)	−	−	−	−	−	−	−	−	−	+	−	−	+	+
*Fusarium oxysporum* (cotton)	−	−	−	−	−	−	−	−	−	+	−	−	+	+
*Fusarium oxysporum* (ATCC 2163)	−	−	−	−	−	−	−	−	−	+	−	−	+	+
*Phytophthora sojae*	−	−	−	−	−	−	−	−	−	+	−	−	−	+
*Ceratocystis paradoxa*	−	−	−	−	−	−	−	−	−	+	−	−	+	+
*Colletotrichum* sp.	−	−	−	−	−	−	−	−	−	+	+	−	+	+
*Rhizopus microsporus*	−	−	−	−	−	−	−	−	−	+	+	−	+	+
*Alternaria alternata*	−	−	−	−	−	−	−	−	−	+	−	−	+	+
*Sphaceloma* sp. (CNPUV 102)	+	−	−	−	−	−	−	−	−	+	−	−	+	+

**Table 3:** Enzymatic activity of bacteria isolated from *Polygala paniculata*.

	Amylase	Cellulase	Protease	Polygalacturonase	Pectate lyase	Lipase	Esterase
PV 46	+	−	+	+	+	+	−
PV 48	−	−	−	−	+	−	−
PV 55	−	−	+	−	−	+	+
PV 110	−	−	−	−	+	−	−
PV 150	+	+	+	+	+	−	−
PV 168	−	−	+	−	−	−	−
WF.RH.72	−	−	−	−	−	−	−
UNK 3	−	−	+	−	−	−	−
GLB 2	−	−	+	−	−	+	+
GLB 10′	−	−	−	−	+	−	−
GLB 10″	−	−	+	−	+	+	−
Roxo 16	−	−	+	−	−	−	−
Roxo 19	−	−	+	−	−	−	−
Roxo 20	−	−	+	−	−	−	−

**Table 4:** Genomic comparisons between *Burkholderia cepacia* COPS and *B. cepacia* strains based on genome comparisons and multilocus sequence analysis.

Genome comparisons						Multilocus sequence analysis		
Strain	GC (%)	ANIm (%)	ANIb (%)	dDDH (%)	Tetra	Locus	Identity (%)	Allele
*Burkholderia cepacia* RB-39	65.91	93.68	91.82	51.00	0.99738	*atp*D	100	*atp*D 235
*Burkholderia cepacia* DDS 7H-2	67.05	92.01	90.20	43.30	0.99559	*glt*B	100	*glt*B 707
*Burkholderia cepacia* DWS 16B-4	67.07	92.01	90.24	43.30	0.99561	*gyr*B	**99.7797**	*gyr*B 1041
*Burkholderia cepacia* LMG 16656	66.88	92.07	90.08	43.60	0.99553	*lep*A	**99.4962**	*lep*A 3
*Burkholderia cepacia* MSMB1302	66.81	**96.22**	**95.19**	67.00	**0.99924**	*pha*C	**99.4805**	*pha*C 279
*Burkholderia cepacia* LK13	66.47	**98.72**	**98.04**	**87.80**	**0.99948**	*rec*A	100	*rec*A 1
*Burkholderia cepacia* NBRC 14074	66.67	**97.56**	**96.86**	77.20	**0.99929**	*trp*B	100	*trp*B 21
*Burkholderia cepacia* NCTC10743	66.60	**97.56**	**96.86**	77.10	**0.99932**			
*Burkholderia cepacia* ATCC 25416	66.58	**97.56**	**96.87**	77.10	**0.99935**			
*Burkholderia cepacia* GG4	66.68	91.30	88.62	36.80	0.99608			
*Burkholderia cepacia* JBK9	66.82	92.35	90.59	45.00	0.99686			
*Burkholderia cepacia* MSMB591WGS	66.43	**98.71**	**98.13**	**87.40**	**0.99932**			
*Burkholderia cepacia* MSMB1224WGS	66.86	**97.94**	**97.23**	**80.70**	0.99892			
*Burkholderia cepacia* LO6	66.99	89.93	86.75	36.20	0.97175			
*Burkholderia* sp. LK4	66.46	**98.78**	**98.05**	**87.80**	**0.99950**			
*Burkholderia reimsis* BE51	66.37	**98.72**	**98.05**	**86.50**	**0.99938**			
*Burkholderia lata* LK27	66.76	**98.53**	**96.91**	**87.90**	**0.99926**			

Bold values based on ANI, dDDH, and TETRA (threshold of ≥ 96%, ≥ 70%, and TETRA > 0.999%, respectively) represent that strains belong to the same genomic species, whereas in multilocus sequence analysis, bold values represent new alleles of housekeeping genes within *Burkholderia* spp.

**Table 5:** Antagonistic activity of *B. cepacia* strain COPS NPE toward phytopathogenic fungi and pathogenic bacteria.

Phytopathogenic fungi			Public health pathogens		
	NPE treatment	Benomyl		GLB2 treatment	
	AI (%)	AI (%)		MIC (*μ*g/mL)	IC50 (*μ*g/mL)
*Moniliophthora perniciosa*	89.32	74.17	*Staphylococcus aureus* ATCC 25923	128	58.36
*Sclerotinia sclerotiorum*	85.53	86.26	*Staphylococcus epidermidis* ATCC 35984	32	22.49
*Fusarium solani*	37.97	85.54	*Enterococcus faecium* ATCC 700221	32	24.11
*Fusarium verticillioides*	27.10	92.79	*Enterococcus faecalis* ATCC 29212	64	26.34
*Fusarium proliferatum*	26.27	92.17	*Acinetobacter baumannii* ATCC 19606	512	298.2
*Fusarium oxysporum* (bean)	15.95	90.58	*Escherichia coli* ATCC 25922	512	103.4
*Fusarium oxysporum* (cotton)	13.21	89.79	*Klebsiella pneumoniae* ATCC 700603	>512^*∗*^	N.D.
*Fusarium oxysporum* (ATCC 2163)	7.17	94.42	*Pseudomonas aeruginosa* ATCC 14502	>512^*∗∗*^	N.D.
*Phytophthora sojae*	61.15	100.00			
*Ceratocystis paradoxa*	82.69	100.00			
*Colletotrichum* sp.	42.27	100.00			
*Rhizopus microsporus*	6.59	26.53			
*Alternaria alternata*	22.53	87.48			
*Sphaceloma* sp. (CNPUV 102)	61.45	27.52			

^*∗*^Partial inhibition at 512 *μ*g/mL (14.09%); ^*∗∗*^partial inhibition at 512 *μ*g/mL (25.36%).

## Data Availability

The data used to support the findings of this study are found in laboratory notebooks of Laboratory of Microbiology and Biomolecules (LaMiB), Department of Morphology and Pathology, Federal University of São Carlos, Brazil.

## Abbreviations

AI: Antagonism index
ANIb: Average nucleotide identity based on BLAST
ANIm: Average nucleotide identity based on MUMmer
Bcc: *Burkholderia cepacia* complex
BHI: Brain-heart infusion agar
CFU: Colony forming units
CLSI: Clinical and Laboratory Standards Institute
dDDH: Digital DNA-DNA hybridization
DMSO: Dimethyl sulfoxide
ISP2: International *Streptomyces* Project medium 2
MALDI-TOF MS: Matrix-assisted laser desorption/ionization time-of-flight mass spectrometry
MIC: Minimum inhibitory concentration
MHCA: Müeller–Hinton cation adjusted
MLSA: Multilocus sequence analysis
MLST: Multilocus sequence typing
NPE: Natural products extract
OD: Optical density
PBS: Phosphate-buffered solution
PDA: Potato dextrose agar
QS: Quorum sensing
ST: Sequence type
TETRA: Tetranucleotide frequency
TSA: Tryptic soy agar
TSB: Tryptic soy broth.

## Supplementary Materials

Supplementary Materials Supplementary Figure 1: consensus tree obtained from a neighbor-joining phylogenetic analysis using the Jukes-Cantor method (bootstrap with 1000 replicates) based on full length 16S rDNA gene of *B. cepacia* COPS strain compared to reference sequences of Bcc members. Supplementary Figure 2: consensus tree obtained from a neighbor-joining phylogenetic analysis using the Jukes-Cantor method (bootstrap with 1000 replicates) based on full length *atp*D gene of *B. cepacia* COPS strain compared to reference sequences of Bcc. Supplementary Figure 3: consensus tree obtained from a neighbor-joining phylogenetic analysis using the Jukes-Cantor method (bootstrap with 1000 replicates) based on full length *glt*B gene of *B. cepacia* COPS strain compared to reference sequences of Bcc. Supplementary Figure 4: consensus tree obtained from a neighbor-joining phylogenetic analysis using the Jukes-Cantor method (bootstrap with 1000 replicates) based on full length *gyr*B gene of *B. cepacia* COPS strain compared to reference sequences of Bcc. Supplementary Figure 5: consensus tree obtained from a neighbor-joining phylogenetic analysis using the Jukes-Cantor method (bootstrap with 1000 replicates) based on full length *lep*A gene of *B. cepacia* COPS strain compared to reference sequences of Bcc. Supplementary Figure 6: consensus tree obtained from a neighbor-joining phylogenetic analysis using the Jukes-Cantor method (bootstrap with 1000 replicates) based on full length *pha*C gene of *B. cepacia* COPS strain compared to reference sequences of Bcc members. Supplementary Figure 7: consensus tree obtained from a neighbor-joining phylogenetic analysis using the Jukes-Cantor method (bootstrap with 1000 replicates) based on full length *rec*A gene of *B. cepacia* COPS strain compared to reference sequences of Bcc. Supplementary Figure 8: consensus tree obtained from a neighbor-joining phylogenetic analysis using the Jukes-Cantor method (bootstrap with 1000 replicates) based on full length *trp*B gene of *B. cepacia* COPS strain compared to reference sequences of Bcc.
