# Supplementary material for: Discovery of a Novel Lineage Burkholderia cepacia ST 1870 Endophytically Isolated from Medicinal Polygala paniculata Which Shows Potent In Vitro Antileishmanial and Antimicrobial Effects
Source: Int J Microbiol. 2021 Feb 17;2021:6618559. doi: 10.1155/2021/6618559 (PMC7904367; doi:10.1155/2021/6618559)
Supplement: Supplementary Materials — Supplementary Figure 1: consensus tree obtained from a neighbor-joining phylogenetic analysis using the Jukes-Cantor method (bootstrap with 1000 replicates) based on full length 16S rDNA gene of B. cepacia COPS strain compared to reference sequences of Bcc members. Supplementary Figure 2: consensus tree obtained from a neighbor-joining phylogenetic analysis using the Jukes-Cantor method (bootstrap with 1000 replicates) based on full length atpD gene of B. cepacia COPS strain compared to reference sequences of Bcc. Supplementary Figure 3: consensus tree obtained from a neighbor-joining phylogenetic analysis using the Jukes-Cantor method (bootstrap with 1000 replicates) based on full length gltB gene of B. cepacia COPS strain compared to reference sequences of Bcc. Supplementary Figure 4: consensus tree obtained from a neighbor-joining phylogenetic analysis using the Jukes-Cantor method (bootstrap with 1000 replicates) based on full length gyrB gene of B. cepacia COPS strain compared to reference sequences of Bcc. Supplementary Figure 5: consensus tree obtained from a neighbor-joining phylogenetic analysis using the Jukes-Cantor method (bootstrap with 1000 replicates) based on full length lepA gene of B. cepacia COPS strain compared to reference sequences of Bcc. Supplementary Figure 6: consensus tree obtained from a neighbor-joining phylogenetic analysis using the Jukes-Cantor method (bootstrap with 1000 replicates) based on full length phaC gene of B. cepacia COPS strain compared to reference sequences of Bcc members. Supplementary Figure 7: consensus tree obtained from a neighbor-joining phylogenetic analysis using the Jukes-Cantor method (bootstrap with 1000 replicates) based on full length recA gene of B. cepacia COPS strain compared to reference sequences of Bcc. Supplementary Figure 8: consensus tree obtained from a neighbor-joining phylogenetic analysis using the Jukes-Cantor method (bootstrap with 1000 replicates) based on full length trpB gene of B. cepacia [file 6618559.f1.docx]

**Novel allele *Burkholderia* *cepacia* endophytically isolated from medicinal *Polygala* *paniculata* shows potent *in vitro* antileishmanial and antimicrobial effects.**

*Felipe de Paula Nogueira Cruz^1,2,3^, Ailton Ferreira de Paula^1^, Camila Tita Nogueira^2^, Paulo Henrique Marques de Andrade^1,2^, Leonardo Maurici Borges^4^, Paulo Teixeira Lacava^1,3^, Ilana Lopes Baratella da Cunha Camargo^5^, Fernanda de Freitas Aníbal ^2,3^, *Cristina Paiva de Sousa^1,3^

Author affiliations:

^1^Laboratory of Microbiology and Biomolecules – LaMiB, Department of Morphology and Pathology, Federal University of São Carlos, Brazil.

^2^Laboratory of Inflammation and Infectious Diseases – LIDI, Federal University of São Carlos, Brazil.

^3^Biotechnology Graduate Program, Federal University of São Carlos, Brazil.

^4^Laboratory of Taxonomy and Plant Evolution – TaxEP, Department of Botany, Federal University of São Carlos, Brazil.

^5^Laboratory of Molecular Epidemiology and Microbiology, Physics Institute of Sao Carlos, University of Sao Paulo - São Carlos - Brazil.

*Correspondence: Felipe de Paula Nogueira Cruz, depaula_fc@outlook.com, +55 11 93021-3487.

**Supplementary information**

**SUPPLEMENTARY FIGURE 1:** Consensus tree obtained from a Neighbor-Joining phylogenetic analysis using the Jukes-Cantor method (bootstrap with 1000 replicates) based on full length 16S rDNA gene of *B. cepacia* COPS strain compared to reference sequences of Bcc members.


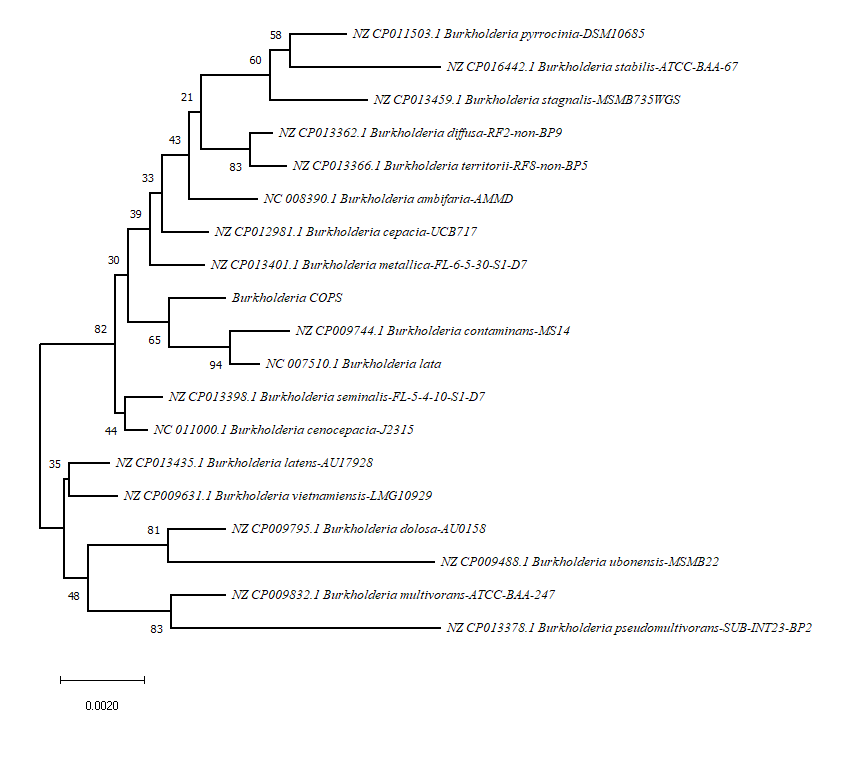


**S1**


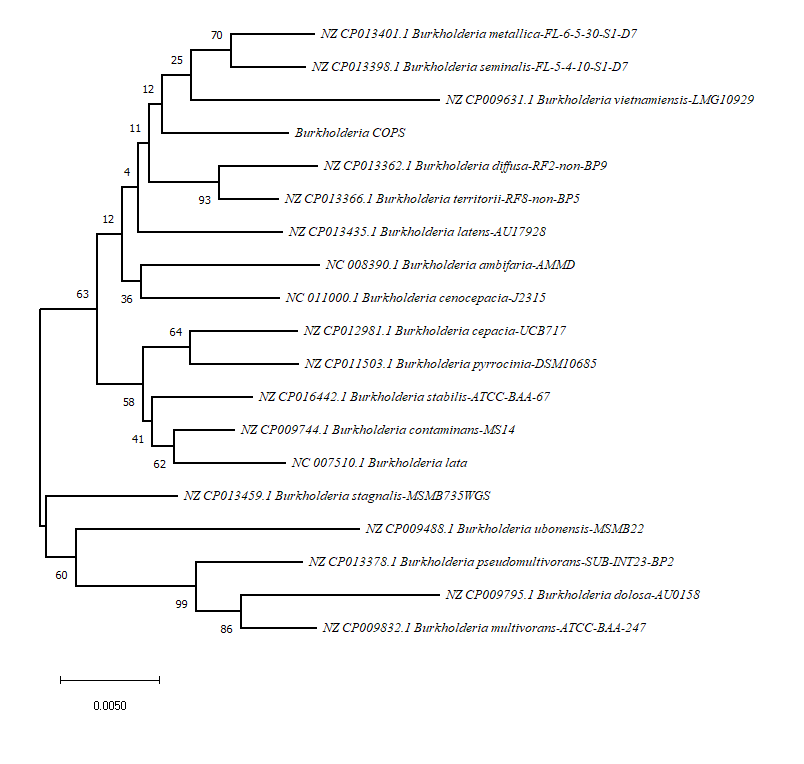
**SUPPLEMENTARY FIGURE 2:** Consensus tree obtained from a Neighbor-Joining phylogenetic analysis using the Jukes-Cantor method (bootstrap with 1000 replicates) based on full length *atp*D gene of *B. cepacia* COPS strain compared to reference sequences of Bcc members.

**S2**


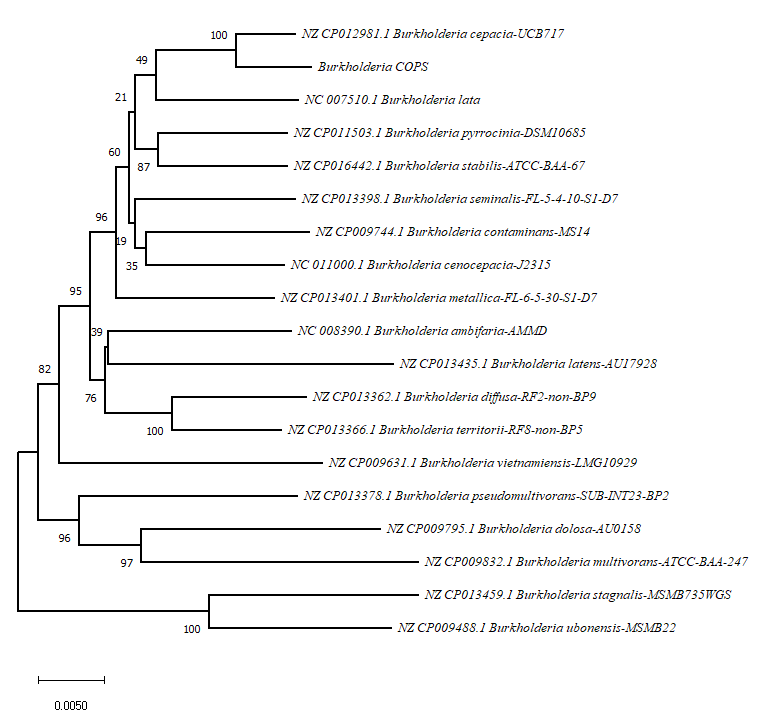
**SUPPLEMENTARY FIGURE 3:** Consensus tree obtained from a Neighbor-Joining phylogenetic analysis using the Jukes-Cantor method (bootstrap with 1000 replicates) based on full length *glt*B gene of *B. cepacia* COPS strain compared to reference sequences of Bcc members.

**S3**


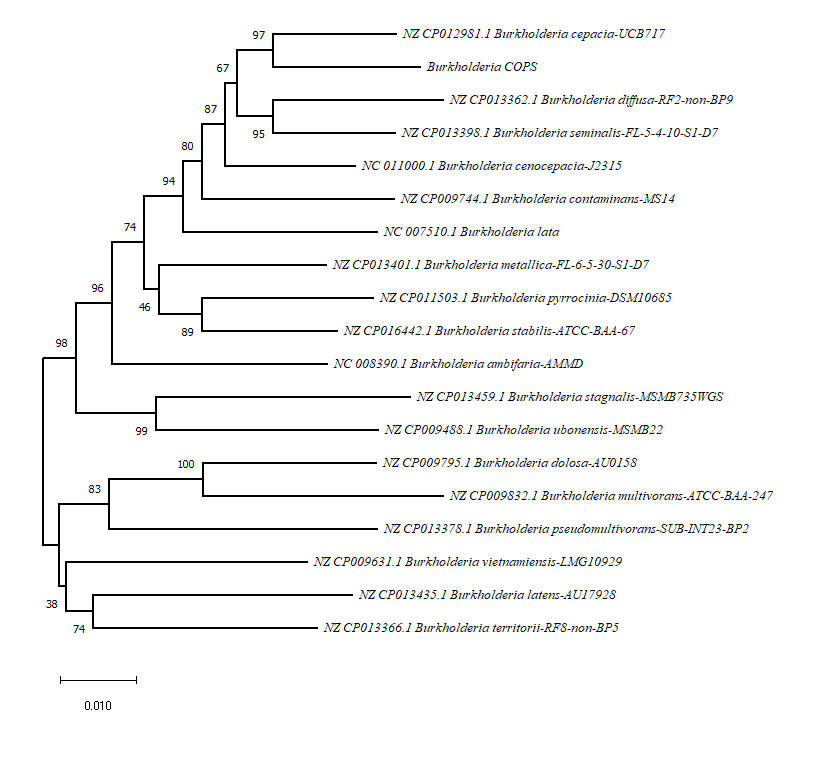
**SUPPLEMENTARY FIGURE 4:** Consensus tree obtained from a Neighbor-Joining phylogenetic analysis using the Jukes-Cantor method (bootstrap with 1000 replicates) based on full length *gyr*B gene of *B. cepacia* COPS strain compared to reference sequences of Bcc members.

**S4**


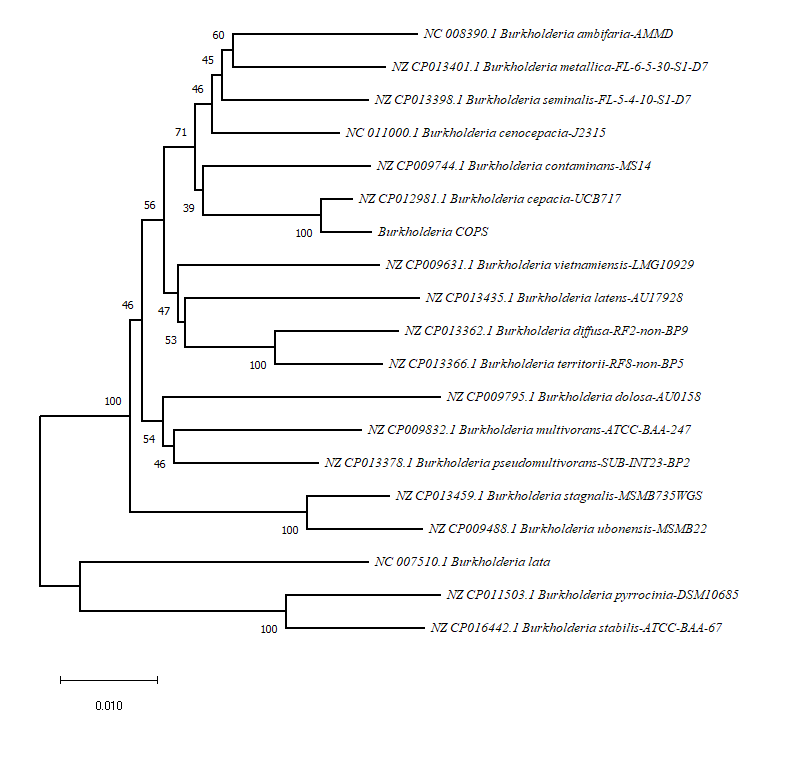
**SUPPLEMENTARY FIGURE 5:** Consensus tree obtained from a Neighbor-Joining phylogenetic analysis using the Jukes-Cantor method (bootstrap with 1000 replicates) based on full length *lep*A gene of *B. cepacia* COPS strain compared to reference sequences of Bcc members.

**S5**


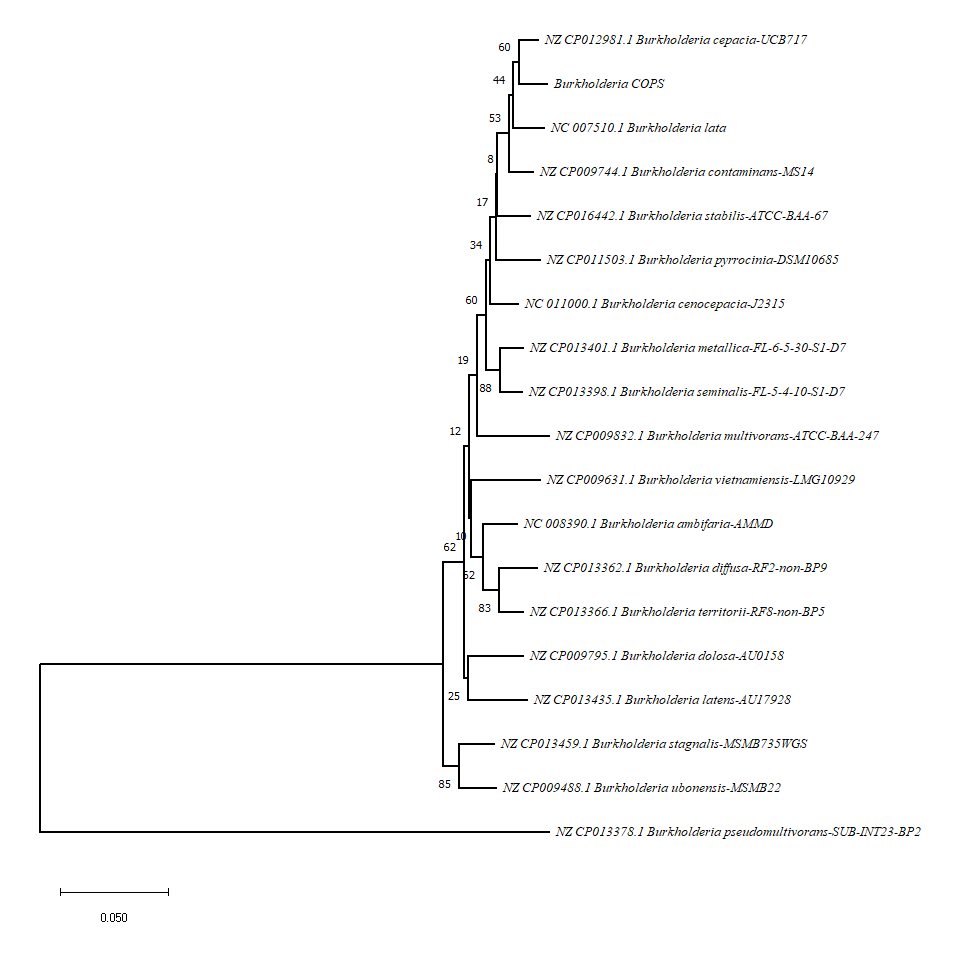
**SUPPLEMENTARY FIGURE 6:** Consensus tree obtained from a Neighbor-Joining phylogenetic analysis using the Jukes-Cantor method (bootstrap with 1000 replicates) based on full length *pha*C gene of *B. cepacia* COPS strain compared to reference sequences of Bcc members.

**S6**


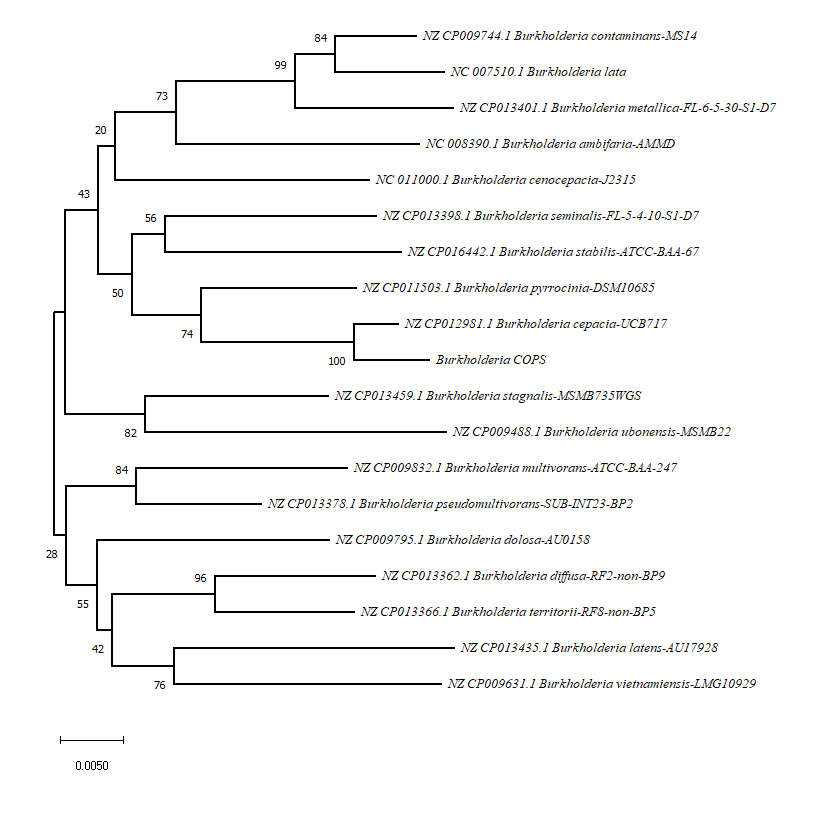
**SUPPLEMENTARY FIGURE 7:** Consensus tree obtained from a Neighbor-Joining phylogenetic analysis using the Jukes-Cantor method (bootstrap with 1000 replicates) based on full length *rec*A gene of *B. cepacia* COPS strain compared to reference sequences of Bcc members.

**S7**


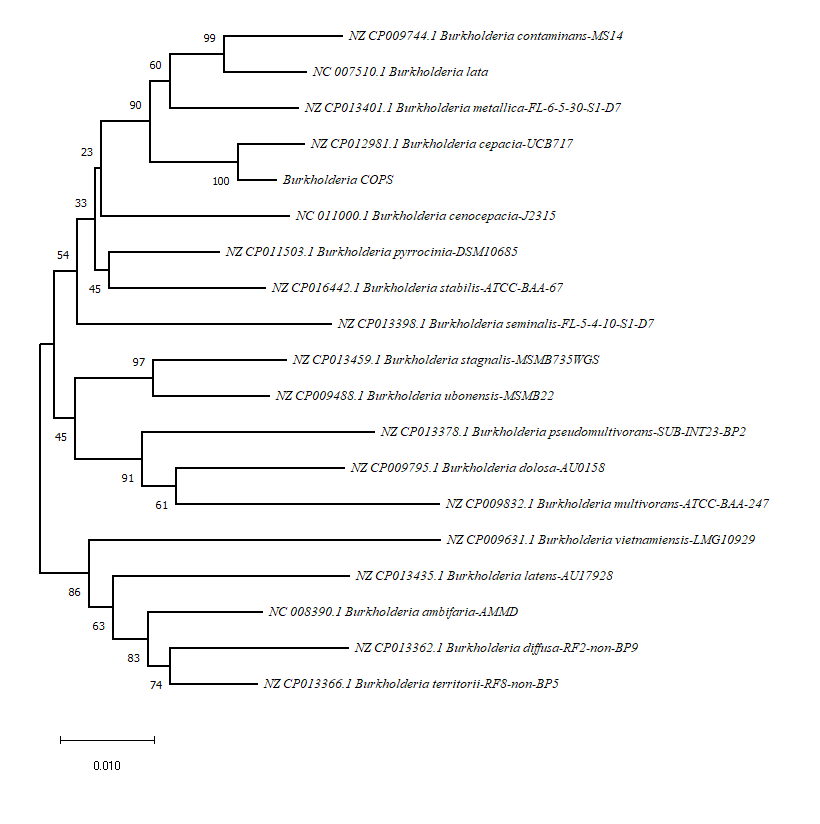
**SUPPLEMENTARY FIGURE 8:** Consensus tree obtained from a Neighbor-Joining phylogenetic analysis using the Jukes-Cantor method (bootstrap with 1000 replicates) based on full length *trp*B gene of *B. cepacia* COPS strain compared to reference sequences of Bcc members.

**S8**
